# Procalcitonin, Interleukin-6 and C-reactive Protein Levels Predict Renal Adverse Outcomes and Mortality in Patients with Acute Type A Aortic Dissection

**DOI:** 10.3389/fsurg.2022.902108

**Published:** 2022-04-28

**Authors:** Xuelian Chen, Jiaojiao Zhou, Miao Fang, Jia Yang, Xin Wang, Siwen Wang, Lichuan Yang

**Affiliations:** ^1^ Division of Nephrology, Department of Medicine, West China Hospital of Sichuan University, Chengdu, China; ^2^ Department of Ultrasound, West China Hospital of Sichuan University, Chengdu, China; ^3^ Department of Orthopedics, Second People’s Hospital of Chengdu, Chengdu, China; ^4^ Department of Pediatric Nephrology, West China Second University Hospital, Sichuan University, Chengdu, China

**Keywords:** acute type A aortic coarctation, inflammatory biomarkers, interleukin-6, C-reactive protein, procalcitonin, adverse renal outcomes, mortality

## Abstract

**Background:**

Acute type A aortic coarctation (AAAD) is a highly deadly and serious life-threatening disease. The purpose of this study was to estimate the predictive value of peak procalcitonin, interleukin-6, and C-reactive protein levels on adverse renal outcomes and mortality in patients undergoing surgery for AAAD.

**Methods:**

Perioperative peak PCT, CRP, and IL-6 levels were retrospectively collected in 331 patients hospitalized with AAAD from 2009 to 2021. The primary endpoints were AKI stage 2–3 and mortality. The receiver operating characteristic (ROC) curves were used to compare the predictive values of peak PCT, CRP, and IL-6 for different clinical outcomes. Multivariable logistic regression analysis was used to find risk factors for AKI and 30-day mortality.

**Results:**

The incidence of AKI stage 2–3 following AAAD was 50.8% (168/331). The 30-day and overall mortality were significantly greater in the AKI 2–3 group than in the AKI 0–1 group (*P* = 0.000). ROC curve analysis showed that peak PCT, with an area under the ROC curve (AUC) of 0.712, was a more accurate predictor of adverse renal outcomes than peak IL-6 and CRP. Multivariable logistic regression analysis revealed that PCT > 0.39 ng/mL was an independent risk factor for AKI stage 2–3. Peak IL-6 > 259 pg/mL was found to be an independent risk factor for 30-day mortality.

**Conclusion:**

In patients with surgery for AAAD, peak PCT provides a well-predictive indicator of AKI stage 2–3 and peak IL-6 indicates a favorable predictor of 30-day mortality.

## Introduction

Acute kidney injury (AKI) is a common complication in hospitalized patients and in intensive care unit (ICU) admissions of critically ill patients (1–3), associated with high morbidity and mortality (4). AKI develops in 30% of patients undergoing cardiac surgery, with approximately 1% of patients requiring dialysis (5). AKI is associated with increased mortality after cardiac surgery (6, 7). In contrast to other heart surgeries, aortic surgery results in a higher incidence of dialysis-requiring acute kidney injury (D-AKI) (8).

Gender, age, body mass index (BMI), obesity, hypertension, renal malperfusion, preoperative serum creatinine (Scr), prolonged cardiopulmonary bypass (CPB) time, postoperative blood transfusion, and sepsis are risk factors for the development of AKI in cardiac surgery (9–12). The pathophysiological mechanisms of AKI associated with cardiac surgery are complex, including the direct inflammatory injury following renal hypoperfusion and ischemia-reperfusion injury (13), with the inflammatory response being central (14).

In addition, the pathogenesis and prognosis of acute type A aortic dissection (AAAD) are also relevant to the inflammatory response (15, 16). A number of inflammatory markers such as interleukin-6 (IL-6), interleukin-10 (IL-10), and C-reactive protein (CRP) have been revealed to be independent risk factors for AKI and mortality in patients after cardiac surgery (17, 18). Inflammatory biomarkers are receiving increasing attention as prognostic indicators in patients after cardiac surgery. The impact of perioperative inflammatory biomarkers on clinical outcomes has been understudied in patients undergoing surgery for AAAD.

Inflammatory biomarker levels during the perioperative period vary continuously with factors such as time, extent of disease, and medical intervention. Peak inflammatory markers may be more predictive than those measured at admission (19). In this study, we postulated that higher peak inflammatory biomarkers would have a predictive value for prognosis in patients with AAAD. The aim of this study was to evaluate the role of peak procalcitonin (PCT), CRP, and IL-6 in predicting renal adverse outcomes and mortality in patients undergoing surgery for AAAD.

## Materials and Methods

### Patients

This retrospective study was approved by the ethics committee of West China Hospital, Sichuan University, and registered at the Chinese Clinical Trial Registry (ChiCTR1900021290). Informed consent was waived given that this was a retrospective study. We reviewed the electronic medical records of adult patients undergoing surgery for AAAD at West China Hospital, Sichuan University, between 2009 and 2021. All participants were diagnosed as AAAD by enhanced computed tomography (CT) or echocardiography. Patients were excluded with the following factors: end-stage renal disease (ESRD) or requiring renal replacement therapy (RRT); post kidney transplantation; death within 24 h after admission to hospital; incomplete data. Finally, the study included 331 patients.

### Data Collection

From the electronic medical record system, we obtained baseline characteristics and perioperative data of the patients. Laboratory data were available from the clinical laboratory of the investigator’s hospital by analysis of venous blood specimens collected on admission. Peak CRP, PCT and IL-6 were defined as the highest levels of CRP, PCT and IL-6 in the perioperative period.

### Outcomes

The primary outcomes were the development of AKI stage 2–3 and mortality. According to the kidney disease: Improving Global Outcomes criteria (KDIGO) (20), patients with ≥200% Scr rise from baseline within 7 days, urine output <0.5 mL/kg/h for more than 12 h or requiring for RRT were assigned to group of AKI stage 2–3. 30-day mortality was defined as death within 30 days after surgery for AAAD. Overall mortality referred to the total number of deaths during the follow-up period.

The second outcomes were acute kidney disease (AKD), requiring for continuous renal replacement therapy (CRRT), Ventilator time, and length of stay (LOS) in hospital.

### Statistical Methods

Continuous variables were presented as means ± standard deviations (SD) or medians (25th, 75th percentile). Categorical variables were presented as percentages. Student’s t-test, chi-square test, or Mann-Whitney U-test were performed to compare differences between groups. The area under the receiver operating characteristic (ROC) curve (AUC) was used to compare the predictive power of PCT, IL-6, and CRP for adverse outcomes. The z-test was applied to verify the difference between the different ROC curves. Multivariable logistic regression analysis was used to identify independent risk factors for the occurrence of AKI stage 2–3 and 30-day mortality. The Hosmer-Lemeshow test was applied to test the goodness of fit of these logistic regression models. A *P* value of <0.05 was considered statistically significant.

All statistical analysis and statistical plots were performed with the SPSS software package, version 26.0 (IBM Corp., Chicago, USA), GraphPad Prism 8.0 software (GraphPad Software, Inc., San Diego, CA, USA) and Medcalc software.

## Results

### Demographic Characteristics of Patients

According to **Table 1**, the prevalence of AKI stage 0–1 and AKI stage 2–3 after surgery in this study was 49.2% (163/331) and 50.8% (168/331), respectively. Preoperative variables such as BMI, hypertension, poor blood pressure control, New York Heart Association (NYHA) III–IV, and liver insufficiency were statistically different between the two groups (*P* < 0.05). Based on laboratory findings, two groups showed statistically significant differences in baseline Scr, urea nitrogen (BUN), uric acid (UA), cystatin C (Cys-C) proteinuria, and low hematocrit levels (<24%). Compared to the AKI stage 0–1 group, the AKI stage 2–3 group had a higher incidence of CPB duration ≥180 min and red blood cell (RBC) transfusion during the procedure for AAAD (*P* < 0.05). Additionally, those with AKI stage 2–3 were more likely to require CRRT, had a higher incidence of AKD, delayed recovery of renal function, and a longer duration of mechanical ventilation than patients with AKI stages 0–1 (*P* < 0.05). Finally, there was a significant difference in 30-day and overall mortality between patients with AKI stage 0–1 and 2–3 (*P* < 0.05).

**Table 1:** Baseline and clinical Characteristics of patients with surgery for AAAD.

Variables	ALL patients (*n* = 331)	AKI stage 0–1 (*n* = 163)	AKI stage 2–3 (*n* = 168)	*P* value^a^
Age, (year)	48 ± 11	47 ± 11	49 ± 11	0.054
Gender				**0.030**
Male	262 (79.2%)	121 (74.2%)	141 (83.9%)	
Female	69 (20.8%)	42 (25.8%)	27 (16.1%)	
BMI, (kg/m^2^)	24.9 ± 3.9	24.3 ± 3.3	25.5 ± 4.3	**0.005**
Smoking	151 (45.6%)	71 (43.6%)	80 (47.6%)	0.458
Drinking	90 (27.2%)	44 (27.0%)	46 (27.4%)	0.937
Medical history				
Hypertension	198 (59.8%)	87 (53.4%)	111 (66.1%)	**0.018**
Poor blood pressure control	83 (25.1%)	32 (19.6%)	51 (30.4%)	**0.024**
COPD	17 (5.1%)	5 (3.1%)	12 (7.1%)	0.093
CKD	21 (6.3%)	7 (4.3%)	14 (8.3%)	0.132
Diabetes mellitus	18 (5.4%)	6 (3.7%)	18 (5.4%)	0.165
Marfan syndrome	16 (4.8%)	7 (4.3%)	9 (5.4%)	0.652
Previous cardiac surgery	21 (6.3%)	16 (9.8%)	5 (3.0%)	**0.011**
Aortic regurgitation	143 (43.2%)	77 (47.1%)	66 (39.3%)	0.144
Lab data				
Baseline Scr, (μmol/L)	82 (65, 104)	77 (64, 94)	88 (68, 113)	**0.001**
BUN, (mmol/L)	6.60 (4.90, 8.95)	5.88 (4.50, 7.80)	6.95 (5.65, 10.57)	**<0.001**
UA, (umol/L)	354 (281, 444)	328 (260, 401)	384 (307, 488)	**<0.001**
Cys-C, (mg/L)	1.32 (0.91, 2.20)	1.00 (0.85, 1.30)	1.97 (1.35, 3.36)	**<0.001**
ALB, (g/L)	37.6 (32.8, 40.7)	38.1 (34.2, 40.8)	36.6 (31.7, 40.6)	0.075
Low hematocrit levels (<24%)	62 (18.7%)	22 (13.5%)	40 (23.8%)	**0.016**
Hematuria	91 (27.5%)	39 (23.9%)	52 (31.0%)	0.152
Proteinuria	136 (41.1%)	49 (30.1%)	87 (51.8%)	**<0.001**
Inflammatory markers				
Peak CRP, (mg/L)	113 (70, 166)	101 (56, 146)	118 (88, 195)	**0.001**
Peak PCT, (ng/mL)	0.83 (0.24, 2.89)	0.41 (0.15, 1.19)	1.51 (0.52–5.83)	**<0.001**
Peak IL-6, (pg/mL)	165.40 (81.46, 373.00)	162.00 (72.84, 331.30)	178.65 (86.84, 429.55)	0.109
Preoperative comorbidities				
Hemorrhagic shock	8 (2.4%)	3 (1.8%)	5 (3.0%)	0.501
Pericardial tamponade	14 (4.2%)	7 (4.3%)	7 (4.2%)	0.954
NYHA III-IV	83 (25.1%)	27 (16.6%)	56 (33.3%)	**<0.001**
Liver insufficiency	47 (14.2%)	14 (8.6%)	33 (19.6%)	**0.004**
Renal malperfusion	95 (28.7%)	41 (25.2%)	54 (32.1%)	0.160
Introperative factors				
Total aortic arch replacement	245 (74.0%)	114 (69.9%)	131 (78%)	0.096
DHCA	81 (24.5%)	36 (22.1%)	45 (26.8%)	0.320
CPB duration ≥180 min	293 (88.5%)	138 (84.7%)	155 (92.3%)	**0.030**
RBC transfusion, (units)	2.0 (0.0, 4.0)	2.0 (0.0, 4.0)	3.0 (0.0, 4.5)	**0.003**
Outcomes				
AKI stage				_
stage 1	81 (24.5%)	_	_	
stage 2	74 (22.4%)	_	_	
stage 3	94 (28.4%)	_	_	
AKD	77 (23.3%)	8 (4.9%)	69 (41.1%)	**<0.001**
CRRT	57 (17.2%)	2 (1.2%)	55 (32.7%)	**<0.001**
Ventilator time, days	4 (2, 7)	3 (1, 4)	6 (4, 10)	**<0.001**
LOS in hospital, days	20 ± 10	24 ± 3	25 ± 4	0.158
30-day mortality	39 (11.8%)	4 (2.5%)	35 (20.8%)	**<0.001**
Overall mortality	55 (16.6%)	8 (4.9%)	47 (28.0%)	**<0.001**
Fellow-up, year	2.70 (0.71, 4.97)	2.76 (1.51, 5.03)	2.09 (0.20, 4.85)	**0.014**

*^a^* *AKI stage 0-1 vs. No AKI stage 2–3.*

*Peak CRP, PCT and IL-6 were defined as the highest levels of CRP, PCT and IL-6 in the perioperative period.*

*Bold values indicate statistically significant (P < 0.05).*

*Continuous variables were presented as mean ± SD or median (25th, 75th percentile). Categorical variables were presented as numbers and percentages.*

*AKI, acute kidney injury; AKD, acute kidney disease; CPB, cardiopulmonary bypass; BMI, body mass index; CKD, chronic kidney disease; COPD, chronic obstructive pulmonary disease; DHCA, Deep hypothermic circulatory arrest; SD, standard deviation; SCr, serum creatinine; Cys-C, cystatin C; ALB, albumin; BUN, blood urea nitrogen; UA, uric acid; PCT, procalcitonin; IL-6, interleukin-6; CRP, C-reactive protein; NYHA, New York Heart Association; RBC, red blood cell; CRRT, continuous renal replacement therapy.*

### Peak CRP, PCT and IL-6 Levels and ROC Analysis

**Figure 1:**
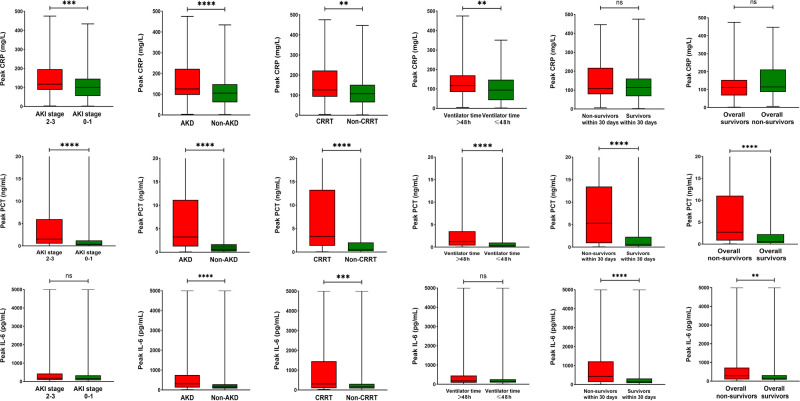
Peak CRP, PCT and IL-6 levels in the different groups according to major clinical outcomes. ^ ^**P < 0.05, ***P < 0.001, ****P < 0.0001, “ns” represents no statistically significant differences (*P* > 0.05). AKI, acute kidney injury; AKD, acute kidney disease; CRRT, continuous renal replacement therapy; PCT, procalcitonin; IL-6, interleukin-6; CRP, C-reactive protein.

**Figure 2:**
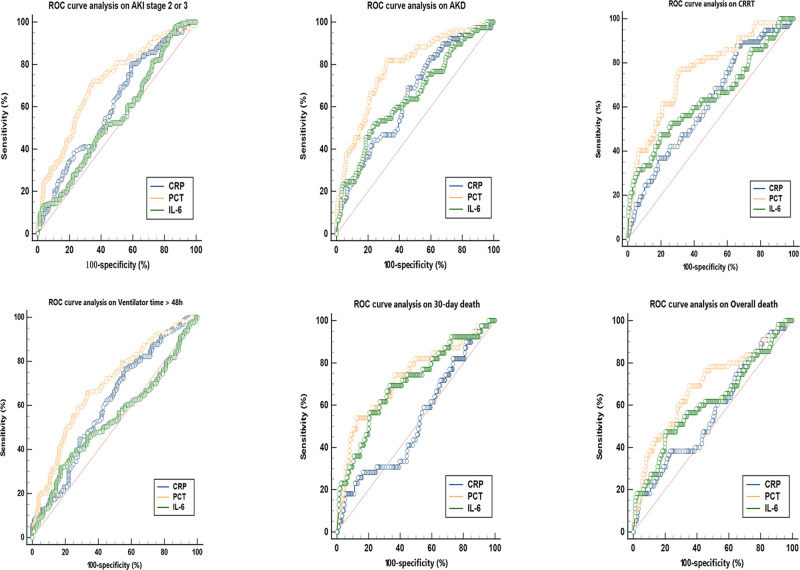
ROC curve analysis on major endpoints.

As presented in **Figure 1**, patients were classified into separate groups based on clinical outcomes in order to assess differences in inflammatory biomarker levels. The study’s study revealed that there was a significant difference in peak CRP and PCT levels, but no difference in IL-6 levels, between the AKI stage 2–3 and 0–1 groups. In comparison to surviving patients, dead patients (both those at 30 days and overall death) had significantly different peak PCT and IL-6 levels, while CRP levels were not statistically different. The peak CRP, PCT, and IL-6 levels were higher in the groups of AKD and CRRT (*P* < 0.05). The ROC curves were used to verify the predictive power of CRP, PCT, and IL-6 on renal outcomes and mortality, and the results were presented in **Table 2**. PCT (AUC, 0.712) showed a better predictive value for AKI stage 2–3 compared to CRP (AUC, 0.607). The AUC of IL-6 for predicting AKI stage 2–3 was 0.551 (0.496–0.605, *P* > 0.05), which was not statistically significant. Among the three inflammatory markers, PCT demonstrated the greatest predictive value for AKD and CRRT, while IL-6 and CRP had similar predictive effects. In predicting 30-day mortality, PCT had a similar predictive value to IL-6, while CRP had no predictive value. In **Figure 2**, the sensitivity and specificity of peak CRP, PCT, and IL-6 in predicting clinical endpoints were shown.

**Table 2:** The AUC (95% CI) values of peak CRP, PCT and IL-6 on major endpoint.

Outcome	CRP (95%CI)	PCT (95%CI)	IL-6 (95%CI)	*P* value^a^	*P* value^b^	*P* value^c^
AKI stage 2–3	0.607 (0.553–0.660)	0.712 (0.660–0.761)	0.551 (0.496–0.605)*	**0.0018**	–	–
AKD	0.651 (0.596–0.702)	0.783 (0.734–0.826)	0.649 (0.595–0.701)	**0.0005**	0.9733	**0.0002**
CRRT	0.621 (0.566–0.673)	0.761 (0.711–0.806)	0.641 (0.587–0.693)	**0.0026**	0.6728	**0.0035**
Ventilator time ≥48 h	0.608 (0.553–0.661)	0.689 (0.636–0.738)	0.536 (0.481–0.591)*	**0.0223**	–	–
30-day death	0.529 (0.474–0.584)*	0.728 (0.677–0.776)	0.703 (0.651–0.752)	–	–	0.6362
Overall death	0.567 (0.512–0.621)	0.688 (0.635–0.738)	0.616 (0.561–0.669)	**0.0180**	0.3876	0.1288

*^a^* *CRP vs. PCT.*

*^b^* *CRP vs.IL-6.*

*^c^* *PCT vsq. IL-6.*

* *AUC was not statistically significant (P > 0.05).*

*ROC, receiver operating characteristic; AUC, area under the ROC curve; PCT, procalcitonin; IL-6, interleukin-6; CRP, C-reactive protein.* *Bold values indicate statistically significant (P < 0.05).*

### Risk Factors of AKI stage 2–3 and 30-day mortality

In **Table 3**, multivariable binary logistic regression analysis suggested that Cys-C (adjusted odds ratio [OR], 4.348, *P* < 0.001) and NYHA III–IV (adjusted OR, 1.985, *P* = 0.034) were independent risk factors for AKI stage 2–3. The second tertile (adjusted OR, 3.444, *P* < 0.001) and the third tertile (adjusted OR, 4.239, *P* < 0.001) of peak PCT were independently associated with AKI stage 2–3 when compared to the lowest tertile. Peak CRP and IL-6 were not found to be independently associated with AKI stage 2–3.

**Table 3:** Logistic regression analysis of risk factors for AKI stage 2–3 and 30-day mortality.

Endpoints	Factors	Adjusted OR (95%CI)	*P* value
AKI stage 2 or 3^a^	**Cys-C, (mg/L)**	4.348 (2.860–6.610)	**<0.001**
	**NYHA III-IV**	1.985 (1.053–3.742)	**0.034**
	**Peak PCT**		
	T1 (<0.39)	1 (reference)	-
	T2 (0.39–1.80)	3.444 (1.789–6.629)	**<0.001**
	T3 (>1.80)	4.239 (2.120–8.476)	**<0.001**
30-day mortality^b^	**NYHA III-IV**	3.659 (1.637–8.179)	**0.002**
	**Ventilator time**	1.054 (1.002–1.109)	**0.042**
	**AKI stage 2–3**	3.633 (1.087–12.149)	**0.036**
	**CRRT**	4.669 (1.966–11.085)	**<0.001**
	**Peak IL-6**		
	T1 (<103)	1 (reference)	–
	T2 (103–259)	2.581 (0.771–8.641)	0.124
	T3 (>259)	4.589 (1.586–13.278)	**0.005**

*^a^* *Logistic regression analysis for AKI stage 2–3 was adjusted for gender, BMI, hypertension, poor blood pressure control in patients with hypertension, previous cardiac surgery, PCT, CRP, BUN, UA, Cys-C, baseline SCr, NYHA III-IV, liver insufficiency, proteinuria, CPB duration ≥180 min, RBC transfusion, and Low haematocrit levels (<24%). (Losmer-Lemeshow test: X^2 ^= 8.571, P = 0.380).*

*^b^Logistic regression analysis for 30-day mortality was adjusted for age, BMI, diabetes, poor blood pressure control in patients with hypertension, NYHA III-IV, aortic regurgitation, PCT, IL-6, ventilator time, Cys-C, AKI stage 2–3, AKD, CRRT, RBC transfusion. (Losmer-Lemeshow test: X^2^ = 5.370, P = 0.717).* *Bold values indicate statistically significant (P < 0.05).*

**Figure 3:**
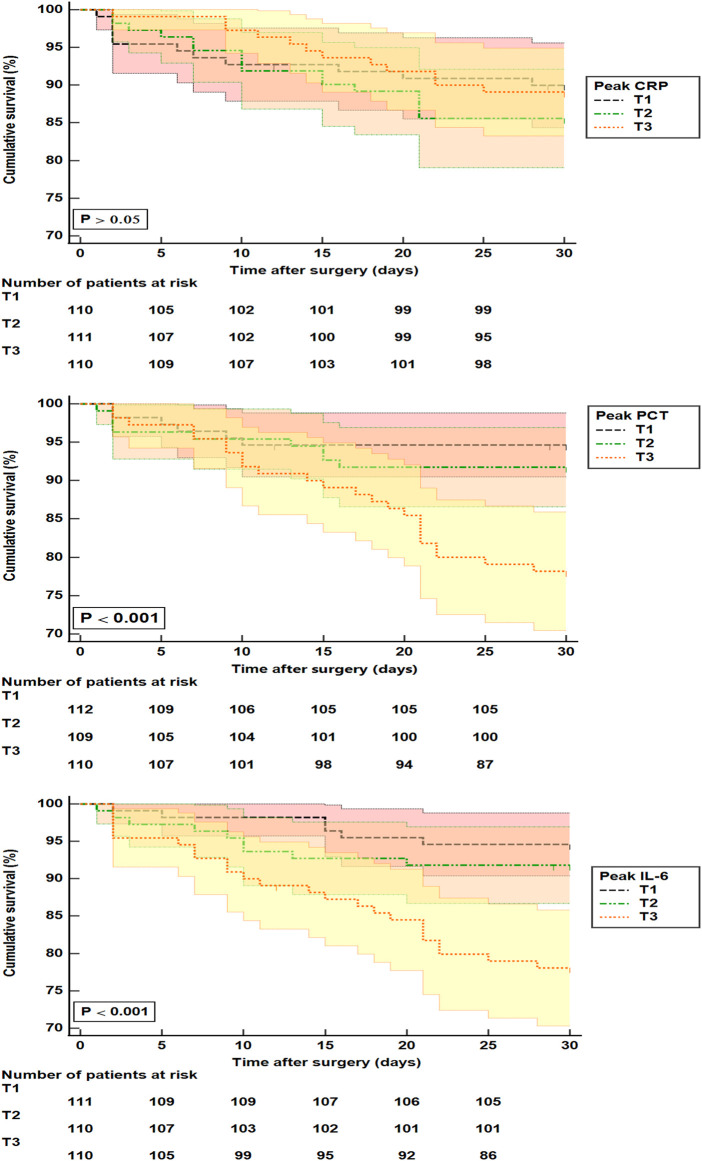
Kaplan-Meier analysis for 30-day mortality in patients with surgery for AAAD. CRP: T1 (<88 mg/L); T2 (88–145 mg/L); T3 (>145 mg/L). PCT: T1 (<0.39 ng/mL); T2 (0.39 –1.80 ng/mL); T3 (>1.80 ng/mL). IL-6: T1 (<103 pg/mL); T2 (103–259 pg/mL); T3 (>259 pg/mL). T, tertile; PCT, procalcitonin; IL-6, interleukin-6; CRP, C-reactive protein.

Kaplan-Meier analysis indicated that patients with higher peak IL-6 and PCT had lower survival rates during 30 days after surgery for AAAD (**Figure 3**). Another logistic regression analysis showed that NYHA III–IV (adjusted OR, 3.659, *P* = 0.002), requiring CRRT (adjusted OR, 4.669, *P* < 0.001), ventilator time (adjusted OR, 1.054, *P* = 0.042), and AKI stage 2–3 (adjusted OR, 3.633, *P* = 0.036) were independently associated with 30-day mortality. While the highest tertile (adjusted OR, 3.633, *P* = 0.036) of peak IL-6 was associated with a higher risk of 30-day mortality compared with the lowest tertile (**Table 3**).

## Discussion

Here, we evaluated the predictive value of peak inflammatory biomarkers for renal adverse outcomes and mortality in patients with AAAD. The findings were as follows: (1) Peak PCT was a better predictor of renal outcomes, including AKI stage 2–3, requiring CRRT, and AKD, compared to CRP and IL-6. A high level of PCT (>0.39 ng/mL) was shown to be an independent risk factor for AKI stage 2–3. (2) Peak PCT and IL-6 were better predictors of mortality than CRP. Peak IL-6 (>259 pg/mL) was an independent risk factor for 30-day mortality. Our study confirmed the association between high levels of PCT and AKI 2–3. In addition, we also noted that PCT levels were higher in patients requiring CRRT and in those with a longer duration of renal injury. A prospective study by Kurtul et al. (21) demonstrated that serum PCT levels at admission were independently associated with the development of contrast-associated acute kidney injury. Clementi et al. (22) and Brocca A et al. (14) both concluded that PCT levels within 48 h after cardiac surgery were predictive of AKI. Liu et al. (23) also suggested that PCT levels were predictive of the development of AKI stage 3. The relationship between cardiac surgery and post-operative AKI and PCT levels is complicated. Trauma, tissue damage, infection, and inflammation are known to cause PCT to remain elevated (24). Because of the systemic inflammatory response syndrome (SIRS) (25), cardiac surgery can lead to elevated serum PCT levels and acute kidney injury (26).Furthermore, the direct cytotoxicity of PCT can exacerbate kidney damage (27). Renal dysfunction could contribute to a decrease in renal clearance of PCT, indirectly causing an increase in PCT levels (28), leading to a vicious cycle. In our analysis, this may be the reason why PCT levels at different time periods can be used as a biomarker for AKI.

CRP represents an independently risk factor for various cardiovascular diseases (29, 30). CRP levels are significantly elevated in patients with AAAD and are related to poor prognosis in patients with AAAD. Schillingeret al. and Wen et al. also concluded that patients with high levels of CRP at admission were at high risk of short-term mortality (18, 31). In the present study, peak CPR levels were higher in patients with adverse renal outcomes, while surprisingly, no differences were found in CRP levels between 30-day and overall survivors and non-survivors. This inconsistency may be due to the fact that the survival of patients with AAAD is also affected by a variety of factors, including age, medical conditions, surgery-related factors, and postoperative complications (32, 33).

Our findings indicated that IL-6 (>259 pg/mL) would be an independent risk factor for 30-day mortality postoperatively. The predictive value of IL-6 and PCT for 30-day mortality and overall mortality after surgery for AAAD was greater in comparison to CRP. Moreover, the risk of 30-day mortality was in a dose-dependent manner related to the PCT and IL-6 levels. Studies reported that IL-6 was a good biomarker to predict mortality after cardiac surgery (14, 22). IL-6 is a pro-inflammatory cytokine that plays a critical role in the initiation and resolution of a variety of inflammation and immunological responses, as well as inducing the synthesis of significant levels of C-reactive protein (34). SIRS after cardiac surgery can be exacerbated by prolonged CPB, cytokinemia, and high IL-6 levels (35, 36). Furthermore, SIRS and IL-6 levels were found to be associated with an increased risk of pneumonia, multiple organ failure, and mortality (37). Remarkably, no direct connection between IL-6 and AKI stage 2–3 was observed in the current investigation. This finding contradicts prior research indicating that increased IL-6 levels following cardiac surgery are significantly associated with an increased risk of AKI (17, 38). It has been claimed that IL-6 expression is associated with the development and severity of AKI and that IL-6 plays a dual function in its induction, aggravating renal injury via a cell-mediated immune response and reducing renal injury by activation of a protective response in the tubular epithelium (39). Since this study relies on retrospective research, a relatively small sample size, and the possibility of bias, these findings should be interpreted with caution. A greater IL-6 level was shown to be associated with AKD and CRRT patients. Brocca et al. also reported a significant increase in IL-6 in patients requiring RRT (14).

### Study Limitations

This study has several limitations. First, this is a retrospective study conducted in a single center with a limited number of patients studied. Clinical data were collected by different people at different times and may be subject to selection bias. Therefore, a prospective large-scale multicenter study is needed to confirm our results. Second, we recorded only peak PCT, IL-6, and CRP levels, not variations in these inflammatory markers. As a result, the prognostic significance of inflammatory marker levels at certain time points (e.g., admission, one day preoperatively, one day postoperatively, seven days postoperatively, etc.) could not be determined. Despite these limitations, it is reasonable to conclude that peak PCT, IL-6, and CRP levels are useful for predicting renal outcomes and mortality.

## Conclusion

This study suggested that elevated perioperative inflammatory biomarkers were associated with postoperative clinical outcomes in patients with AAAD. Peak PCT could be a useful inflammatory biomarker for predicting AKI stages 2–3 in patients with AAAD surgery, while peak IL-6 could be a reliable predictive indicator of 30-day mortality.

## Data Availability

The datasets were obtained from the database of West China Hospital. The datasets generated and analyzed during the current study are available from the corresponding author on reasonable request.
